# Editorial: Molecular Pathophysiology of Diabetes-Related Organ Injury

**DOI:** 10.3389/fmed.2022.875444

**Published:** 2022-03-18

**Authors:** Tingting Zhao, Qin Zhou

**Affiliations:** ^1^Beijing Key Laboratory for Immune-Mediated Inflammatory Diseases, Institute of Clinical Medical Sciences, China-Japan Friendship Hospital, Beijing, China; ^2^Department of Nephrology, The First Affiliated Hospital, Sun Yat-sen University, Guangzhou, China; ^3^National Health Commission Key Laboratory of Nephrology, Guangdong Provincial Key Laboratory of Nephrology, The First Affiliated Hospital, Sun Yat-sen University, Guangzhou, China

**Keywords:** diabetes mellitus, diabetic kidney disease, diabetic cardiomyopathy, molecular pathophysiology, diagnostic biomarkers and treatment

## Introduction

The prevalence of diabetes mellitus is increasing rapidly around the world. This disease accompanies by a large variety of multiple organs injuries. For example, diabetic kidney disease (DKD) is affecting one in three patients with diabetes, and cardiovascular (CV) disease is the leading cause of death for these patients. However, the pathophysiology is complex and heterogenic, and the therapeutic strategies are limited. The goal of this Research Topic is to collect the recent advances on pathophysiology, a timely diagnosis and prompt treatment with the final aim to retard the progression of diabetes-related organs injury. Sixty-seven contributions covering the listed Research Topics have submitted to this special issue.

## Advances in Adipose Tissues Affected by Insulin-Deficient Diabetes Mellitus

Since adipose tissue is recognized to be an endocrine organ, its role in the pathology of diabetes has aroused great concern. Flenkenthaler et al. provided novel pathophysiologic insights of insulin-deficient diabetes in adipose tissue depots. Multi-omics analysis of mesenteric visceral adipose tissue and subcutaneous adipose tissue of a diabetic, insulin-deficient pig model indicates regionally different metabolic adaptations to overcome energy stress caused by reduced glucose utilization in MIDY adipocytes.

## Reviews in Molecular Pathophysiology of Diabetic Kidney Disease and Cardiomyopathy

Heart and kidney, both as the higher energy-demanding tissues in the body, shared the common pathogenesis, such as metabolic abnormalities, mitochondrial damage and dysfunction, oxidative stress, inflammation, epigenetic factors, and others. Salvatore et al. summarized the major pathophysiological changes and the underlying mechanisms leading to myocardial remodeling and cardiac functional derangement in diabetic cardiomyopathy. As a conclusion, the main driving force of the pathological processes specific of diabetic cardiomyopathy is hyperglycemia, a factor centrally placed among multiple interwoven pathways involving complex cellular and molecular perturbations, which affect both myocardial structure and function.

Similarly, Chang et al. commented the high glucose transport state and local relative oxygen deficiency (primary and secondary) in proximal tubular might be the initial factors of tubular damage, while excessive mitochondria damages and ROS production are important contributors to the further damage of PTs in DKD. Abnormalities in hemodynamics, glucose and lipid metabolism, mitochondria, oxidative stress, inflammation, and many other factors interact with each other and form a vicious circle, leading to the renal tubular dysfunctions. This review systematically presented the mechanism of the pathogenesis of tubular injury in different stages in the development of DKD, which provide clues to develop specific treatments to prevent and delay the tubular injury in DKD.

### Lipid Metabolism in Diabetic Kidney Disease and Cardiomyopathy

Under normal condition, fatty acids are the main energy source of the kidney and heart. In diabetics, the utilization of fatty acid is changed to glycolysis and lipid accumulation. Given the critical role of proximal tubular injury in developing diabetes, Chang et al. and Wang et al., both reviewed that tubular lipotoxicity may occur before mitochondrial dysfunction and is an earlier event in DKD; tubular lipotoxicity may be an indicator for early prediction of DKD.

Ma et al. investigated the susceptibility of 8 polymorphisms in *ApoB* and *PCSK9* genes to DKD in 575 Chinese patients with type 2 diabetes mellitus vs. 653 controls. They indicate that *ApoB* gene is a candidate gene for DKD in Chinese patients with type 2 diabetes mellitus, and its association with hypertension may mediate the association of ApoB gene with DKD.

### Advances in Circular RNAs as Diagnostic Biomarkers and Therapeutic Targets in Kidney Disease

Circular RNAs (circRNAs) are a new type of non-coding RNA molecules which regulate gene expression through epigenetic modifications that have attracted more and more attention in recent years. Yu et al. introduce the biogenesis and biological function of circRNAs, and focus on state-of-art regarding circRNAs as novel biomarkers and therapeutic targets in common kidney diseases. They summarized the roles of circRNAs in the diagnosis and prognosis prediction on renal cell carcinoma, acute kidney injury, and glomerular diseases, including DKD. It is worth mentioning that dysregulated circRNAs, especially those from exosomes, are potential biomarkers in the pathogenesis and progression of DKD. Targeting these circRNAs may provide new therapeutic targets for the clinical treatment of DKD.

### Advances in Herb Medicine Treating Diabetic Kidney Disease and Cardiomyopathy

Herb medicine is important in current therapies. The ebook included three articles of herb medicine treating DKD and cardiomyopathy. Hu et al. reported Tangdshen Formula, a Chinese herbal medicine for the treatment of DKD may alleviate myocardial fibrosis in KKAy mouse models by inhibiting TGF-β/Smad and Wnt/β-catenin signaling pathways, which proves that there is a common pathogenesis of DKD and cardiomyopathy. Gao et al. sdemonstrated Qing-Re-Xiao-Zheng Formula modulates gut microbiota and inhibits inflammation in mice with DKD, and the underlying mechanism of which was proposed to have an involvement of the inhibition of LPS/TLR4/NF-κB pathway. Liu et al. reviewed the active compounds and therapeutic target of *Tripterygium wilfordii Hook. f*. (TWHF) in attenuating proteinuria in diabetic nephropathy. TWHF widely used to treat DKD in China. Tripterygium wilfordii polyglycosides, triptolide, and Celastrol are the effective medicine against proteinuria and kidney injury in diabetic nephropathy. Their Mechanisms are including anti-inflammation, antioxidation, anti-fibrosis, activating autophagy, and anti-podocyte apoptosis, *via* several mechanisms.

## Conclusion

In conclusion, these studies improved our understanding on molecular pathophysiology of diabetes-related organ injury. Potential therapeutic methods and targets are also proposed for the future development of effective therapies to the prevention and treatment of the major clinical problem of diabetic kidney disease and cardiomyopathy.

## Author Contributions

TZ and QZ reviewed the papers included in the Research Topic of *Molecular Pathophysiology of Diabetes-Related Organ Injury* and summarized in the Editorial their main findings, together with a commentary on the current knowledge about diabetic kidney disease and cardiomyopathy. All authors contributed to the article and approved the submitted version.

## Funding

This study was supported by the National Natural Science Foundation of China [No: 81973627] and Beijing Natural Science Foundation [No: 7212195].

## Conflict of Interest

The authors declare that the research was conducted in the absence of any commercial or financial relationships that could be construed as a potential conflict of interest.

## Publisher's Note

All claims expressed in this article are solely those of the authors and do not necessarily represent those of their affiliated organizations, or those of the publisher, the editors and the reviewers. Any product that may be evaluated in this article, or claim that may be made by its manufacturer, is not guaranteed or endorsed by the publisher.

